# Analysis of late toxicity in nasopharyngeal carcinoma patients treated with intensity modulated radiation therapy

**DOI:** 10.1186/s13014-014-0326-z

**Published:** 2015-01-13

**Authors:** YingJie Zheng, Fei Han, WeiWei Xiao, YanQun Xiang, LiXia Lu, XiaoWu Deng, NianJi Cui, Chong Zhao

**Affiliations:** Department of Radiotherapy, Beijing Chaoyang Hospital, Affiliated to Capital Medical University, Beijing, 100020 PR China; Department of Radiation Oncology, Sun Yat-sen University Cancer Center, State Key Laboratory of Oncology in Southern China; Collaborative Innovation Center for Cancer Medicine, Guangzhou, 510060 PR China; Department of Nasopharyngeal Carcinoma, Sun Yat-Sen University Cancer Center, State Key Laboratory of Oncology in Southern China; Collaborative Innovation Center for Cancer Medicine, Guangzhou, 510060 PR China

**Keywords:** Nasopharyngeal neoplasm/radiotherapy, Intensity-modulated radiotherapy, Radiation injury, Late toxicity

## Abstract

**Purpose:**

To observe the late toxicities in nasopharyngeal carcinoma (NPC) patients who achieved long-term survival after intensity modulated radiation therapy (IMRT).

**Methods:**

208 untreated NPC patients who received IMRT and survived more than five years with locoregional disease control and no metastasis were evaluated in this study. The prescription dose to the gross target volume of nasopharynx (GTVnx), positive neck lymph nodes (GTVnd), clinical target volume 1 (CTV1) and 2 (CTV2) was 68Gy/30f, 60-66Gy/30f, 60 Gy/30f and 54Gy/30f, respectively. The nasopharynx and upper neck targets were irradiated using IMRT, and the lower neck and supraclavicular fossae targets were irradiated using the half-beam technique with conventional irradiation. The late toxicities were evaluated according to the LENT/SOMA criteria of 1995.

**Results:**

The median follow-up time was 78 months (60–96 months). The occurrence rates of cervical subcutaneous fibrosis, hearing loss, skin dystrophy, xerostomia, trismus, temporal lobe injury, cranial nerve damage, cataract, and brain stem injury induced by radiotherapy were 89.9%, 67.8%, 47.6%, 40.9%, 7.21%, 4.33%, 2.88%, 1.44%, and 0.48%, respectively. No spinal cord injury and mandible damage were found. Grade 3–4 late injuries were observed as follows: 1 (0.48%) skin dystrophy, 4 (1.92%) cervical subcutaneous fibrosis, 2 (0.96%) hearing loss, 2 (0.96%) cranial nerve palsy, and 1 (0.48%) temporal lobe necrosis. No grade 3–4 late injuries occurred in parotid, temporomandibular joints and eyes. Xerostomia decreased gradually over time and then showed only slight changes after 4 years. The change in the incisor distance stabilised by 1 year after RT, however, the incidence of hearing loss, skin dystrophy, subcutaneous fibrosis and nervous system injuries increased over time after RT.

**Conclusion:**

The late injuries in most NPC patients who had long-term survivals after IMRT are alleviated. Within the 5 years of follow-up, we found xerostomia decreased gradually; The change in the incisor distance stabilised by 1 year after RT; while hearing loss, nervous system injuries increased over time after RT.

## Background

Nasopharyngeal cancer (NPC) is one of the most important head and neck cancers in China. More than 50% of NPC patients treated with two-dimensional radiation therapy (2-D RT) achieved long-term survival [1]. However, late adverse events are obvious in these surviving NPC patients and greatly affect their quality of life [2]. For example, 30%-40% of the surviving NPC patients had late injuries of the neural system [2], and 36% of the surviving NPC patients had grade 3–4 non-neural system injuries [3], which resulted in 1-3% potential lethal damage in these patients [4,5]. Treatments to alleviate late injuries after RT and to improve the tumour control rate are a primary focus of current studies. Intensity modulated radiation therapy (IMRT), which was developed to improve the local tumour control rate and the quality of life, has been widely adapted in NPC treatment in recent decades. Compared with 2-D RT, IMRT effectively lowers the exposure dose of normal tissues around the tumour, such as the parotid glands and temporomandibular joints (TMJs) [6-9]. IMRT not only clearly alleviates acute RT injuries, such as xerostomia, during the middle and later periods of RT [10] but also remarkably reduces the degree of late injuries more than six months after RT [6-12]. However, many reports regarding the late injuries of NPC patients after IMRT had short follow-up periods. We observed the long-term late toxicities in NPC patients after IMRT in the present study.

## Methods

### General information

From February 2003 to March 2007, 208 untreated NPC patients who received IMRT in the Department of Radiation Oncology of Sun Yat-Sen University Cancer Centre and who survived more than five years with locoregional disease control and no metastasis were included in this study. This patient group was composed of 167 male and 41 female patients. The median age was 42 years (range: 13–73 years). According to the Union for International Cancer Control (UICC) 2002 staging criteria, 14 patients were stage I, 82 patients were stage II, 86 patients were stage III, 25 patients were stage IVa, and 1 patient was stage IVb (Table 1).

**Table 1:** **T/N stage distribution of 208 patients**

**Stage**	**T1**	**T2**	**T3**	**T4**	**Total**
N0	14	35	18	4	71
N1	10	37	26	15	88
N2	3	25	14	6	48
N3	1	0	0	0	1
Total	28	97	58	25	208

### The exposure dose of the target region and the organs at-risk

All of the patients received definitive external irradiation. The nasopharynx and upper neck targets were irradiated using the simultaneous modulated accelerated radiation therapy (SMART) boost IMRT technique, and the lower neck and supraclavicular fossae targets were irradiated using the half-beam technique with conventional 2-D irradiation.

The patient immobilisation, CT simulation and target volume delineation details have been described previously by Zhao [10] and Xiao [13]. The brainstem, spinal cord, optic nerves, optic chiasm, pituitary, temporal lobes, lens, parotids, TMJs and mandible were contoured as organs at risk (OARs).

The target volume prescription doses of the gross tumour volume of the nasopharynx (GTVnx), the gross tumour volume of the involved lymph nodes (GTVnd), clinical target volume 1 (CTV1, the area from 0.5 cm to 1.0 cm outside the GTV that involves the potential sites of local infiltration) and clinical target volume 2 (CTV2, the margin from 0.5 cm to 1.0 cm around CTV1 and the lymph node draining area (Levels II, III, and IV)) were 68 Gy, 60–66 Gy, 60 Gy and 54 Gy, respectively. In total, 30 fractions were administered at 1 fraction daily, 5 days/week. The maximum doses to OARs, which were restricted to prevent exceeding their tolerance doses, were as follows: 56 Gy for the brainstem; 45 Gy for the spinal cord; 60 Gy for the temporal lobes; 5 Gy for the lens; 50 Gy for the optic nerves, chiasm, TMJs, and mandible; and 40 Gy for the parotids. This plan was approved upon meeting the following criteria: (1) the prescribed dose encompassed at least 95% of the target volume, (2) no greater than 1% of the GTVnx could receive ≤93% of the prescribed dose, (3) the maximum dose point was located in the GTVnx, and (4) the isodoses were normalised to the maximum dose, with the chosen prescription isodose for the GTVnx equalling at least 80% of the maximum dose. For the critical organs with functional subunits organised in a series, the dose to 5% of the volume (D5) could not exceed their tolerance doses, and the maximum dose delivered to 1 mL of the cervical segment of spinal cord could not exceed 45 Gy. For the critical organs with functional subunits organised in parallel, the dose constraints to 33% of the volume (D33) were less than their tolerance doses [10,13]. The actual exposure doses of targets and OARs are presented in Table 2.

**Table 2:** **The actual exposure doses of targets and parts of organs at risk**

**Target volume**		**Minimal dose mean**	**Maximum dose mean**	**Average dose mean**	**Average fractional dose mean**	**V95 mean**	**Volume**	**D33/D5 mean**
		**(Gy)**	**(Gy)**	**(Gy)**	**(Gy)**	**(%)**		**(Gy)**
GTVnx		63.42	78.34	71.99	2.40	99.38		
GTVndL		59.07	71.53	71.53	2.38	99.73		
GTVndR		59.16	73.42	66.47	2.22	99.93		
CTV1		54.16	77.38	67.59	2.25	99.60		
CTV2		37.54	76.68	60.83	2.03	98.22		
OAR								
Brainstem		5.47	50.47	21.99	1.54		5%	46.13
Spinal cord		7.59	37.23	24.74	1.32		1 cc	39.45
Temporal lobes	L	1.73	61.79	12.49	0.49		33%	14.76
	R	2.00	61.95	12.42	0.51			15.27
Parotids	L	14.68	58.30	31.62	1.18		33%	35.38
	R	15.00	59.09	32.26	1.22			36.60
Temporomandibular joints	L	19.34	47.54	29.22	1.15		33%	34.51
	R	19.75	46.51	29.38	1.16			34.84
Optical nerves	L	6.66	34.00	16.15	1.09		5%	32.58
	R	6.88	33.52	16.27	1.10			32.99
Optic chiasma		9.79	27.50	16.75	0.90		5%	26.93

### Chemotherapy

Of 208 patients, 127 received radiotherapy alone, 81 received chemoradiotherapy. Of 81 patients, 45 received concurrent chemotherapy, 11 received induction and concurrent chemotherapy, 9 received concurrent and adjuvant chemotherapy, 5 received induction, concurrent and adjuvant chemotherapy, and 11 received induction or adjuvant chemotherapy. The chemotherapy regimens were primarily based on cisplatin or on cisplatin and 5-fluorouracil (5-Fu).

### Follow-up and late toxicity assessment

All patients were followed up at 3 months post-treatment. The patients were then followed up every 3 months until 3 years post-treatment and then once a year thereafter. Complete physical examination and indirect laryngoscopy or fiber optic pharyngorhinoscopy were performed every follow-up visit. Blood and biochemistry profiles, chest radiography, abdominal ultrasonography, and magnetic resonance imaging (MRI) or computed tomography (CT) scan of the nasopharynx and cervical region were routine elements of the assessment. Further investigations were arranged as indicated. Late toxicity was evaluated according to the LENT/SOMA (late effect of normal tissues/subjective, objective, management, analytic) criteria of 1995 [14]. Radiation-induced cranial nerve injuries were defined as symptoms or signs of cranial nerve damage without tumour recurrence. If the patient presented cranial nerve injuries before radiotherapy and fully recovered after treatments, then the recurrence of symptoms or signs of cranial nerve damage were recorded as radiation-induced cranial nerve injury. If the patient only partially recovered, then exacerbations of symptoms or signs were also recorded.

### Statistical methods

SPSS 13.0 (Chicago, IL) software was used for the statistical analysis. Logistic regression was adopted for multiple-factor analysis. P values < 0.05 were considered as statistically significant.

## Results

### Follow-up period and late toxic effect occurrence

**Figure 1:**
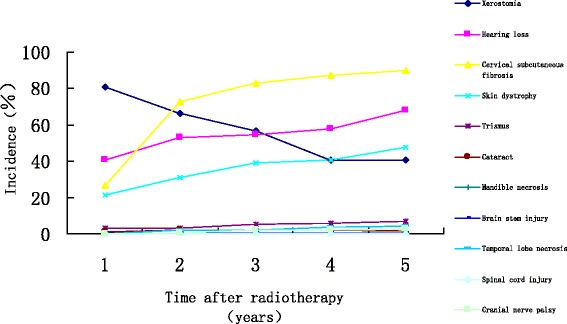
**The incidence of late injuries within 5 years after radiotherapy.**

The follow-up period began on the last day of radiotherapy, and the last follow up was 31 March 2012. The median follow-up time was 78 months (range: 60–96 months). Of 208 patients, 180 (86.5%), 13 (6.25%), 5 (2.4%), 2 (0.48%) and 4 (1.92%) patients experienced one or more late toxic effects within 12 months, 13–24 months, 25–36 months, 37–48 months and 49–60 months after receiving treatments, respectively. The most common late injury was cervical subcutaneous fibrosis, with an occurrence rate of 89.9%. Hearing loss, skin dystrophy and xerostomia had occurrence rates of 67.8%, 47.6% and 40.9%, respectively. Trismus, temporal lobe injury, cranial nerve damage, cataract and brain stem injury had occurrence rates of 7.21%, 4.33%, 2.88%, 1.44% and 0.48%, respectively. No spinal cord injuries or mandible injuries were found. Xerostomia decreased gradually over time. In contrast, hearing loss, skin dystrophy, and cervical subcutaneous fibrosis increased gradually over time (Figure 1).

### Late toxicity of the nervous system

In total, 16 patients (7.69%) had late injuries of the nervous system, including 1 (0.48%) with brain stem injury, 6 (2.88%) with cranial nerve damage and 9 (4.33%) with temporal lobe injury (Table 3). Two patients presented grade 4 injuries of post-cranial nerves. Patients with brain injuries exhibited varying degrees of unsteady gait, limb weakness, memory loss and limb numbness. The patient that developed a brain stem injury at 1 year was diagnosed with stage T4 with the GTVnx next to the brain stem. Cranial nerve damage was found in 1, 2, 1 and 2 patients in years 2, 3, 4 and 5 after RT, respectively. Temporal lobe injury was found in 4, 2, 2 and 1 patients in years 2, 3, 4 and 5 after RT, respectively. The distribution of the T stages is shown in Table 4.

**Table 3:** **Grades of late injuries of nervous system**

**Total number 208**						
**Late injury**	**Total**	**Total %**	**Distribution of patients**			
			**1**	**2**	**3**	**4**
	**n**		**Grade**	**Grade**	**Grade**	**Grade**
Brain stem	1	0.48	0	1	0	0
Temporal lobe	9	4.33	0	8	**1**	0
Cranial nerve	6	2.88	0	4	0	2
Spinal cord	0	0	0	0	0	0

**Table 4:** **The distribution of T stage of patients who had late nervous system injuries**

	**Late nerves system injuries**				
**Stage**	**Brain stem**	**Temporal lobe**	**Cranial nerve**		**Total**
			**Front**	**Post**	
T1				1	1
T2		1		1	2
T3		4	3		7
T4	1	4	1		6
Total	1	9	4	2	16

### Non-nervous system toxicity

Most late injuries were assessed as grades 0–1. In rare cases, grade 3–4 late injuries were observed as follows: 1 skin injury (0.48%), 4 subcutaneous soft tissue injuries that were severe in duration (1.92%), and 2 hearing injuries (0.96%). No grade 3–4 injuries were observed in the parotids, TMJs or eyes, and no late mandible injuries were observed (Table 5).

**Table 5:** **Distribution of late injuries occurrence in non-nervous system**

**Total number 208**						
**Late injury**	**Total**	**Total %**	**Distribution of patients**			
			**1**	**2**	**3**	**4**
	**n**		**Grade**	**Grade**	**Grade**	**Grade**
Parotid	85	40.9	75	10	0	0
Ear	141	67.8	98	41	2	0
Subcutaneous soft tissue	187	89.9	139	44	4	0
Skin	99	47.6	81	17	1	0
Temporomandibular joints	15	7.21	10	5	0	0
Eye	3	1.44	2	1	0	0
Mandible	0	0	0	0	0	0

**Figure 2:**
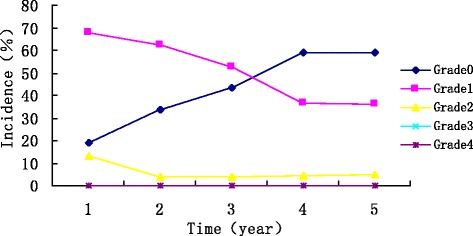
**The incidence and grade of xerostomia.**

Xerostomia was recorded in 80.8%, 66.3%, 56%, 40.9% and 40.9% of patients within 1, 2, 3, 4 and 5 years after RT, respectively. The incidence decreased gradually over time and then stabilised within the 4 years after RT. No grade 3–4 xerostomia was found (Figure 2).

**Figure 3:**
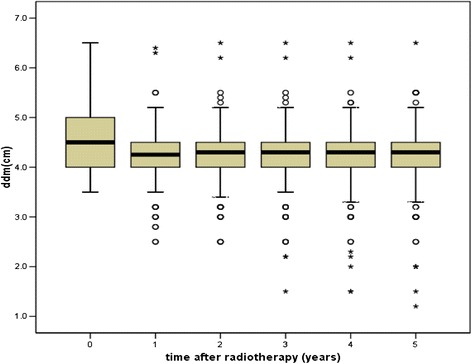
**The change of incisor distance of 208 patients 1-5 year after radiotherapy.**

The incisor distance stabilised by 1 year after RT in all patients. Trismus was recorded in 3.37%, 3.37%, 5.29%, 5.77% and 7.21% patients within 1, 2, 3, 4 and 5 years after RT, respectively. No grade 3–4 trismus developed. The mean incisor distance of 208 patients was 4.54 ± 0.5 cm before radiotherapy and decreased to 4.3 ± 0.6 cm, 4.3 ± 0.6 cm, 4.3 ± 0.7 cm, 4.2 ± 0.7 cm and 4.2 ± 0.7 cm by years 1–5 after radiotherapy, respectively (Figure 3).

### Multifactorial analysis of late injuries

Logistic multifactorial analysis was used to analyse the independent risk factors, which included gender, age, T stage, clinical stage and chemotherapy of late injuries after IMRT, for NPC patients. Hearing loss was related to chemotherapy and age (p < 0.05). Xerostomia was associated with chemotherapy (p < 0.05) (Table 6).

**Table 6:** **Multiple-factor analysis of late injuries**

			**HR (95% CI; P)**		
	**Salivary glands**	**Ears**	**TMJS**	**Skin**	**Subcutaneous soft tissue**
Gender	0.911 (0.439-1.890)	1.015 (0.983-1.049)	1.766 (0.508-6.141)	0.759 (0.376-1. 53)	6.046 (0.775-47.153)
	P > 0.05	P > 0.05	P > 0.05	P > 0.05	P > 0.05
Age	0.987 (0.959-1.017)	3.311 (1.322-8.288)	1.035 (0.983-1.089)	0.994 (0.967-1.022)	1.005 (0.957-1.054)
	P > 0.05	P = 0.011	P > 0.05	P > 0.05	P > 0.05
Stage (I-II vs III-IV)	1.455 (0.673-3.143)	1.919 (0.833-4.42)	2.511 (0.462-13.656)	0.720 (0.336-1.54)	2.081 (0.596-7.267)
	P > 0.05	P > 0.05	P > 0.05	P > 0.05	P > 0.05
T Stage (T1-2vs T3-4)	0.977 (0.454-2.101)	0.926 (0.400-2.144)	2.496 (0.469-13.280)	1.759 (0.831-3.727)	0.776 (0.230-2.618)
	P > 0.05	P > 0.05	P > 0.05	P > 0.05	P > 0.05
Radiotherapy vs Chemoradiotherapy	2.249 (1.186-4.265)	4.012 (1.839-8.749)	0.681 (0.211-2.203)	1.282 (0.682-2.409)	3.823 (0.998-14.649)
	P = 0.013	P = 0.000	P > 0.05	P > 0.05	P = 0.050

## Discussion

For the anatomic site specificity of nasopharyngeal carcinoma, the irradiation field of traditional RT usually involved many normal tissues, including most salivary glands, temporomandibular joints, optical nerves, ears, temporal lobes, and the spinal cord. However, the radical tumour dose largely exceeded the tolerance dose of normal tissues. Cui [15] reported that the mean doses of the optical nerves, temporal lobes, TMJs, and middle ears all were over 63 Gy and that some were even higher than 69 Gy, with a prescription dose of 70 Gy GTVnx in conventional RT. These high doses resulted in various late adverse events, including hearing loss, xerostomia, and trismus, and greatly affected the patient’s quality of life.

IMRT allows the accurate irradiation of tumours and better protects OARs. This technique adopts an inverse therapy planning system, optimises the prescription dose of the set target volume and the restricted dose of organs at risk, makes the distribution of the high dose curve suited to the shape of the tumour target volume, and clearly reduces the irradiation dose and volume of nearby OARs by adjusting the radial intensity. With the prescription doses of GTVnx, GTVnd, CTV1 and CTV2 of 68 Gy, 60 Gy to 66 Gy, 60 Gy and 54 Gy, respectively, the mean irradiation doses reached 71.99 Gy, 66.47 Gy to 71.53 Gy, 67.59 Gy and 60.83 Gy, respectively, which raised the irradiation dose of the target volume compared with conventional RT. Additionally, the mean fractional irradiation dose also reached 2.03 Gy to 2.4 Gy, while the mean doses of nearby OARs, including the optic nerves, optic chiasma, parotids, TMJs, brainstem and spinal cord, were 16 Gy, 17 Gy, 32 Gy, 29 Gy, 12 Gy, 22 Gy and 25 Gy, which were only one-fourth to one-half of the conventional RT doses. The mean doses of D5 and D33 were also clearly below the tolerance doses, with the mean fractions only ranging from 0.49 Gy to 1.54 Gy (Table 2).

Studies regarding the late complications of 2-D RT and IMRT from different radiotherapeutic centres greatly varied. We selected some of the reports (Tables 7 and 8) with many cases, a longer follow-up time (primarily >4 years) and a consistent grading system to compare their findings.

**Table 7:** **The study reports of late complications occurrence after conventional RT**

	**Xerostomia**	**Ears**	**Subcutaneous soft tissue**	**Skin reaction**	**Trismus**	**Mandible**	**Eyes**	**Brain**	**Spinal cord**	**Cranial nerve**
Sanguineti [5] 1954-92		≥G3 2.6%	≥G3 4.2%	≥G3 1.1%	≥G3 2.9%	≥G3 2.6%		≥G3 1.1%	≥G3 2.4%	≥G3 4.5%
N = 378										
Perez [4] 1956-86			≥G3 10%			≥G3 2%		≥G3 1%		
N = 143										
Yeh [16] 1983-98	90.3%	54%	25%		12%			6%	21%	
N = 849		≥G3 12.4%								
Lee [24] 1994-97		≥G3 8.0%	≥G3 6.5%	≥G3 0.3%	≥G3 0.9%	≥G3 0.6%				≥G3 0.3%
N = 325										
Sumitsawan [3] 2003-05	97.5%	82.5%	62.5%	56%	41.5%		5.5%	7.0%		6.5%
N = 200	≥G3 2.5%	≥G3 36%	≥G3 4%	≥G3 1.5%	≥G3 3%		≥G3 0.5%	≥G3 1.5%		
Kong [2]	99.7%	67.0%	89.0%	81.2%	28.3%	0.6%	12.0%	1.2%	9.8%	31.8%
N = 336	≥G3 23.5%	≥G3 22.0%	≥G3 23.6%	≥G3 16.4%	≥G3 3.3%					
This study N = 208	40.9%	67.8%	89.9%	47.6%	7.21%	0%	1.44%	4.80%	0%	2.88%
	≥G3 0%	≥G3 0.96%	≥G3 1.9%	≥G3 0.48%	≥G3 0%	≥G3 0%	≥G3 0%	≥G3 0.48%	≥G3 0%	≥G3 0.96%

**Table 8:** **The study reports of late complications occurrence after IMRT**

	**Xerostomia**	**Ears**	**Subcutaneous Soft tissue**	**Skin reaction**	**Trismus**	**Mandible**	**Eyes**	**Brain**	**Spinal cord**	**Cranial nerve**
Xiao [13] 2001-05	61.8%	91.2%	94.1%	35.3%	7.4%	0%	1.5%	17.6%		4.4%
N = 68 MFU* = 54 m	≥G3 0%	≥G3 0%	≥G3 1.5%	≥G3 0%	≥G3 0%	≥G3 0%	≥G3 0%	≥G3 0%		≥G3 0%
Chen YY [23] 2001-04					5.7%					
N = 211 MFU* = 48 m					≥G3 0%					
Su [18] 2001-08								15.4%		
N = 259 MFU* = 40 m										
Zhou [17] 2003-06								16%		
N = 305 MFU* = 48.9 m										
Wang [21] 2003-06	≥G3 2.7%	≥G3 0%	≥G3 0.14%	≥G3 0.3%	≥G3 0.7%		≥G3 0%	≥G3 0.14%		
N = 695 MFU* = 66.4 m										
Wu [20] 2006-09	≥G3 0%		≥G3 10%			≥G3 2%		≥G3 1%		
N = 249 MFU* = 54.1 m										
This study 2003–07 N = 208 MFU* = 78 m	40.9%	67.8%	89.9%	47.6%	7.21%	0%	1.44%	4.80%	0%	2.88%
	≥G3 0%	≥G3 0.96%	≥G3 1.9%	≥G3 0.48%	≥G3 0%	≥G3 0%	≥G3 0%	≥G3 0.48%	≥G3 0%	≥G3 0.96%

*MFU: Median follow-up.

The occurrence rate of late injuries in the nervous system after conventional RT in NPC patients was 13.5%-42.8% [2,3,16], the rate of late injuries in the nervous system over grade 3 was 1%-4.5% [3-5]. The stage T3 or T4 NPC patients always required the additional administration of 6–8 Gy to the skull base or to the intracranium. After an irradiation dose of 70 Gy to the nasopharynx, the irradiation dose of the temporal lobes and cranial nerves on both sides of the cavernous sinus reached over 70 Gy, which lead to a greatly increased risk of injury. Of 200 NPC patients with more than 5 years survival after conventional RT, Sumitsawan [3] reported that 7% had brain injuries and 1.5% had severe injuries. Kong’s [2] study showed that late injuries of the spinal cord and of the cranial nerve affected up to 9.8% and 31.8% of patients, respectively, within 5 years after 2-D RT. In our research, 83 patients (40%) were classified as stage T3 or T4. Although the mean irradiation dose of nasopharynx reached 72 Gy, no late spinal injuries occurred, 1 (0.48%) brainstem injury occurred, 6 (2.88%) cranial nerve injuries occurred and 9 (4.33%) temporal lobe injuries occurred. Most patients with temporal lobe injuries were stage T3 or T4 of disease, which might have resulted from the high dose of RT to the temporal lobes because of large tumour invasion to the skull base or to the intracranium. Zhou [17] and Su [18] conducted long-term follow-up studies regarding radiation-induced temporal lobe injury in patients with NPC after IMRT. Both studies showed that the temporal lobe injuries were related to the T stage, which was similar to our results. However, the reported incidence of temporal lobe injury was 16% in Zhou’s study [17] and 15.4% in Su’s study [18], which included 305 and 259 NPC patients, respectively, and 5 years of follow-up. These reported incidence rates were higher than the incidence rates reported in our present study. These results may differ because our study excluded some patients who had recurrence or metastasis; thus, the T3-4 cases were less than 40%. In addition, Zhou’s [17] study showed that the median latency of temporal lobe injury detection in NPC patients after IMRT was 36.85 months with 5 years of follow-up. Moreover, Su’s [18] study showed that the median latency of temporal lobe injury detection in NPC patients after IMRT was 30 months and that the longest latency of temporal lobe necrosis was 56 months. Both studies suggested that the incidence of temporal lobe injury increased gradually over time after IMRT, which was similar to the findings of our study. Although the incidence rates of temporal lobe and cranial nerve damage were relatively low in the present study, the incidence rates increasing year by year in the 5 years of follow-up.

One patient with a grade 2 brainstem injury and stage T4 (cavernous sinus) was included in our study. For this patient, the highest irradiation dose of the brainstem was 54.54 Gy, with a mean dose of 28.79 Gy, which suggested that the injury might have resulted from incidentally high sensitivity of the brainstem to irradiation.

The highest occurrence rate (57% to 99.7%) of late complications after NPC conventional therapy was xerostomia [2,3,16]. Kong [2] reported that 99.7% of NPC patients had varying degrees of xerostomia and that 23.5% had grade 3–4 xerostomia after conventional RT. To achieve a nasopharynx tumour dose of 70 Gy in conventional therapy, the dose of most parotids was greater than 60 Gy. Cooper [19] reported that more than a 50 Gy dose to the parotids would result in irreversible injuries and lead to long-term xerostomia. Hsiung [11] compared the extent of injuries to the parotids after IMRT and conventional RT by measuring the maximum excretion rate (MER) of saliva after stimulating the parotids in treated NPC patients. The results showed that the MER of parotids in patients with IMRT recovered by more than 25% after 6 months in 64.5% of patients who received a mean dose of 43.9 Gy (33.2–58.8 Gy). Kwong [7] evaluated parotid function in early phase NPC patients after IMRT and found that the basic flow of parotids recovered by more than 25% in 60% of patients who received a mean dose of 38.8 Gy (32–46.1 Gy). The above two studies regarding parotid function after IMRT confirmed that parotid function could recover gradually when the irradiation dose was below 50 Gy. In our study, the mean irradiation dose of one-third of the bilateral parotid volume (D33) was approximately 36 Gy, and the mean dose was below 33 Gy. During the 5 years of follow-up, no grade 3 or grade 4 xerostomia was presented. Although the incidence of xerostomia in the first year after IMRT was 80.8%, the symptoms gradually alleviated decreased to 40.9% during the 4th year and then tended to be stable. Figure 2 shows that grade 1–2 xerostomia decreased gradually, whereas grade 0 xerostomia increased during the first 4 years of the follow-up period after IMRT and then showed only slight changes after the 4th year. Moreover, primarily grade 1 reduced to grade 0 xerostomia occurred 2 to 4 years after RT. This result suggested that IMRT could clearly facilitate salivary gland protection over time. Some long-term follow-up (more than 4 years) studies examined late toxicity effects in patients with NPC after IMRT [13,20,21]. Xiao [13] reported xerostomia incidence and development trends that were similar to our xerostomia incidence and development trends. These authors showed that the extent of xerostomia was clearly alleviated by 3 to 12 months after IMRT and that grade 1 back to 0 xerostomia cases were primarily observed at 1 year after treatment. Because their observations ceased during the 4th year after RT, the period at which the variation stabilised was not displayed. Wu [20] also reported that the severity of xerostomia decreased over time; however, no grade 3–4 xerostomia could be found at 2 years after treatment. Wang’s [21] study showed that xerostomia could be relieved to the lowest degree within 3–6 months after IMRT and that the condition showed only slight changes after 1 year. This result differed somewhat from the above two studies and from our present study. This difference might be attributable to the presence of grade 3 xerostomia (2.5%-2.7%) 1–5 years after RT in Wang’s [21] research.

Trismus, which is induced by the high irradiation dose of bilateral TMJs in conventional RT, was also a common complication in NPC patients with long-term survival after conventional RT, with occurrence rates of 12%-41.5% overall and 2.9%-3.3% for grade 3 or 4 trismus [2,3,5,16]. Chen M [22] analysed the incisor distance of 352 NPC patients before conventional RT and at 6 months after RT. The occurrence rate of trismus was 58.5% and the rate of grade 3 or grade 4 trismus was 7.1%, which was much higher than that of our study. In the present study, the D33 of TMJs was below 35 Gy, and the mean dose was below 30 Gy, which highly protected TMJ function. Trismus was identified in 7.21% of patients with 5 years of follow-up, and no grade 3 or grade 4 trismus was observed. This result was similar to Chen YY’s [23] and Xiao’s [13] reports using IMRT, in which 5.7% and 7.4% of NPC patients had grade 1–2 trismus after IMRT with more than 4 years of follow-up, respectively, and no severe trismus was presented. Our analysis also demonstrated that the change in the incisor distance of patients stabilised by 1 year after RT. This variation trend was also similar to that described by Chen YY [23] and by Xiao [13].

Hearing loss was also a common complication in NPC patients after conventional RT. The occurrence rate for hearing loss ranged from 54% to 82.5% in all types of clinical reports [2,3,5,16,24]. Although the occurrence rates of hearing loss in studies by Yeh [16] and by Kong [2] were similar to this study, the occurrence rates of over grade 3 hearing loss were 12.4% and 22%, respectively. Sumitsawan’s [3] study reported that the occurrence rate of over grade 3 hearing loss was as high as 36%, while only a 0.96% occurrence rate of over grade 3 hearing loss was found in this study, which indicated that the occurrence rate of hearing loss was significantly improved by IMRT. This result was similar to that reported by Xiao [13]; although the incidence rate of grade 1–2 hearing loss was 91.2%, no grade 3–4 hearing loss was observed. Our study also showed that the incidence of hearing loss increased gradually during the 5 years of follow-up after IMRT, which suggested that hearing loss presented gradually over time after RT and that once hearing loss occurred, it could not be recovered.

In addition, this study showed that the late toxicities of skin and subcutaneous tissues all gradually increased over time after RT. Grade 3–4 skin dystrophy and subcutaneous fibrosis were less frequent compared with those reported by conventional RT studies [2-5,16,24]. However, in the present study, lower neck and supraclavicular fossae were irradiated using the half-beam technique with conventional RT. The data regarding the late toxicities of skin and subcutaneous induced by IMRT could not be presented objectively.

The risk factors of late injuries after RT in NPC patients include age, clinical stage and chemotherapy [3,16,25], suggesting that patients with locoregionally advanced disease would tend to develop late injuries after RT. In Zhou’s [17] study using IMRT, the categorisation of T stages was also an independent predictor of temporal lobe injury by multivariate analysis. However, in our study, multifactorial analysis showed that most late non-nervous system injuries of NPC patients receiving IMRT had no significant correlation with age, gender, clinical staging, T stage or chemotherapy; however, xerostomia and hearing loss were related to chemotherapy and age. This result may be associated with the low incidence of late injury, particularly for conditions over grade 3. We did not perform multifactorial analysis for the late toxicity of the nervous system because the incidence rates of brain stem injury (0.48%), cranial nerve damage (2.88%) and temporal lobe injury (4.33%) in this study were too low.

## Conclusion

In summary, IMRT exhibited advantages for reducing most late toxicities of NPC patients with long-term survival. Through the 5 years follow-up after IMRT, we found xerostomia could be eased or gradually returned to normal over time and that trend showed only slight changes after the 4 years. The change in the incisor distance of patients stabilised by 1 year after RT, while hearing loss, skin dystrophy, subcutaneous fibrosis and nervous system injuries increased gradually over time. However, our study selected the patients who survive more than five years, and patients with recurrence or metastasis were excluded; thus, these results may be biased to some degree, particularly for late toxicities of the nervous system.

In addition, this study did not perform dosimetric analysis, and we will further study the relationship between sensitive organs and the dosimetry of NPC patients with IMRT.

## Abbreviations

NPC: Nasopharyngeal carcinoma
RT: Radiotherapy
2-D RT: Two-dimensional radiation therapy
IMRT: Intensity modulated radiation therapy
UICC: Union for International Cancer Control
SMART: Simultaneous modulated accelerated radiation therapy
TMJ: Temporomandibular joints
OARs: Organs at risk
GTVnx: Gross target volume of nasopharynx
GTVnd: Gross target volume of positive neck lymph nodes
CTV: Clinical target volume
D5: The dose to 5% of the volume
D33: The dose to 33% of the volume
LENT/SOMA: Late effect of normal tissues/subjective, objective, management, analytic
MFU: Median follow-up
